# Great Gerbils (*Rhombomys opimus*) in Central Asia Are Spreading to Higher Latitudes and Altitudes

**DOI:** 10.1002/ece3.70517

**Published:** 2024-11-11

**Authors:** Xuan Liu, Li Xu, Jianghua Zheng, Jun Lin, Xuan Li, Liang Liu, Ruikang Tian, Chen Mu

**Affiliations:** ^1^ College of Geography and Remote Sensing Sciences Xinjiang University Urumqi China; ^2^ Xinjiang Key Laboratory of Oasis Ecology Xinjiang University Urumqi China; ^3^ Xinjiang Uygur Autonomous Region Locust and Rodent Prediction Forecasting and Prevention Center Station Urumqi China; ^4^ Prairie Station of Animal Husbandry Department in Xinjiang Urumqi China

**Keywords:** changes in spatial distribution, climate change, migration paths, multi‐model integration, *Rhombomys opimus*

## Abstract

The great gerbil ( *Rhombomys opimus* ) is a gregarious rodent in Central Asia and is one of the major pests found in desert forest and grassland areas. The distribution changes and migration routes of *R. opimus* in Central Asia under climate change remain unexplored. This study employed multi‐model ensemble, correlation analysis, jackknife method, and minimum cumulative resistance (MCR) model to simulate the potential habitat of *R. opimus* under current and future (2030 and 2050) climate scenarios and estimate its possible migration routes. The results indicate that the ensemble model integrating Random Forest (RF), Gradient Boosting Machine (GBM), and Maximum Entropy Model (MaxEnt) performed best within the present climate context. The model predicted the potential distribution of *R. opimus* in Central Asia with an area under the curve (AUC) of 0.986 and a True Skill Statistic (TSS) of 0.899, demonstrating excellent statistical accuracy and spatial performance. Under future climate scenarios, northern Xinjiang and southeastern Kazakhstan will remain the core areas of *R. opimus* distribution. However, the optimal habitat region will expand relative to the current one. This expansion will increase with the rising CO_2_ emission levels and over time, potentially enlarging the suitable area by up to 39.49 × 10^4^ km^2^. In terms of spatial distribution, the suitable habitat for *R. opimus* is shifting toward higher latitudes and elevations. For specific migration routes, *R. opimus* tends to favor paths through farmland and grassland. This study can provide guidance for managing and controlling *R. opimus* under future climate change scenarios.

## Graphical abstract

This paper focuses on the distributional changes and migration paths of greater gerbils in Central Asia under different climatic scenarios, with the aim of supporting regional wildlife management.

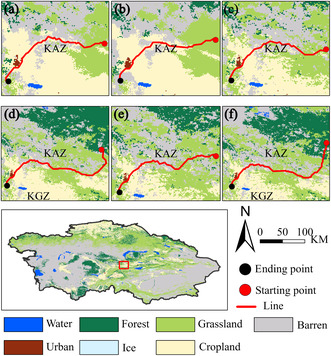

## 1 Introduction

Global changes, represented by climate change, have profound impacts on ecosystem stability and species richness aspects (Fei et al. 2017). As global warming intensifies, climate change poses severe challenges to the survival of flora and fauna (Mori, Furukawa, and Sasaki 2013; Weiskopf et al. 2020). These challenges are evident in population fluctuations (Dullinger et al. 2012; Wan et al. 2022), habitat alterations (An et al. 2023; Niskanen et al. 2019), changes in interspecies relationships (Blois et al. 2013), and shifts in distribution ranges (Nisin, Sreeram, and Paul Sreeram 2023). Additionally, different land use types create diverse geographical landscapes (Bürgi, Östlund, and Mladenoff 2017; Ellis 2021). These landscapes directly impact species' spatial distribution and migration routes (Boisvert‐Marsh and Blois 2021; Powers and Jetz 2019; Semenchuk et al. 2022). Therefore, studying the effects of climate change on species' geographical distribution and analyzing migration routes of suitable habitats in conjunction with land use types are essential for developing effective management strategies.

Central Asia ranks among the world's major regions for oasis agriculture and landscapes, characterized by significant water resource changes and fragile oasis ecosystems. It is highly sensitive to global changes, making it a hotspot and significantly impacted regions for climate change (Zhong et al. 2022). The *Rhombomys opimus* is a typical inhabitant of desert forests and steppes in Central Asia, living in family clusters (Hamidi, Mohammadi, and Ghassemi‐Khademi 2021; Wen et al. 2020). Its impacts are multifaceted. On one hand, the extensive burrowing activity of *R. opimus* significantly reduces surface vegetation cover (Wen et al. 2016), severely disrupting the healthy growth of desert vegetation, leading to the degradation of biological soil crusts, accelerating desertification, and causing continuous ecological deterioration (Prakash and Ghosh 1975). On the other hand, *R. opimus* serves as the primary host of the plague bacterium in Central Asia (Ji et al. 2021). Therefore, accurately predicting the distribution and migration of *R. opimus* can provide scientific and reliable insights for relevant institutions, aiding in the timely development of control measures to mitigate the rodent's destructive impact on desert ecosystems, reduce losses for local residents, and enhance regional disease prevention and early warning capabilities.

Ecological niche models (ENMs), also known as species distribution models (SDMs), use species occurrence data and environmental factors to predict the potential geographical distribution of species (Phillips, Anderson, and Schapire 2006; Sillero et al. 2021). Recently, a growing number of ENMs are now used to assess the effects of environmental and climate changes on species and ecosystems. Currently, the most frequently utilized models for species distribution include generalized linear models (GLM) (Gorosito, Marziali Bermúdez, and Busch 2018), Classification and Regression Trees (CART) (Vayssières, Plant, and Allen‐Diaz 2000), Maximum Entropy Models (MaxEnt) (Phillips, Anderson, and Schapire 2006), BIOCLIM models (Booth et al. 2014), and Random Forest (RF) models (Zhang et al. 2019). Due to differences in principles and algorithms, every model has unique strengths and weaknesses. If input data changes, the performance of each model can become unstable (Beery et al. 2021). To enhance prediction accuracy and address the uncertainty and potential low reliability of single models, more and more researchers are now focusing on ensemble modeling (Ahmad Suhaimi, Blair, and Jarvis 2021; Koshkina et al. 2017; Pacifici et al. 2019). Thuiller and colleagues proposed the first computational framework for integrating multiple SDMs (Thuiller et al. 2009), BIOMOD (Biodiversity Modeling), and released an updated version (BIOMOD2) in 2016. This platform facilitates the combination of results from multiple individual models.

Current research on rodents using SDMs primarily focuses on forecasting climate change impacts on their distribution with single models (An et al. 2023; Perkins‐Taylor and Frey 2020). However, the selection of the optimal model and the inconsistencies between different single models remain unresolved. Meanwhile, current studies often compare the geographical distribution of *R. opimus* under present and future climate conditions to analyze changes in its suitable habitats (Wen et al. 2022), but the specific migration routes of *R. opimus* in Central Asia still require further investigation.

Therefore, this study integrates regional characteristics and considers factors influencing *R. opimus* habitat selection, such as topography, soil properties, vegetation factors and cover, climate conditions, and human activities. We used nine SDMs: RF, MaxEnt, GLM, Gradient Boosting Machine (GBM), Classification and Regression Tree (CART), Artificial Neural Network (ANN), Surface Range Envelope (SRE), Flexible Discriminant Analysis (FDA), and Multiple Adaptive Regression Splines (MARS). We explored the optimal ensemble model for forecasting suitable habitats of *R. opimus* , analyzed habitat changes, and simulated migration routes based on land use types. This study aims to: (1) to determine the optimal SDM for *R. opimus* in Central Asia; (2) to explore the similarities and differences in the geographical distribution of appropriate habitats for *R. opimus* under various future climate conditions; and (3) to elucidate the spatial changes and migration routes of *R. opimus* under climate change.

## 2 Materials and Methods

### 2.1 Study Areas and Species Occurrence Sites

**FIGURE 1:**
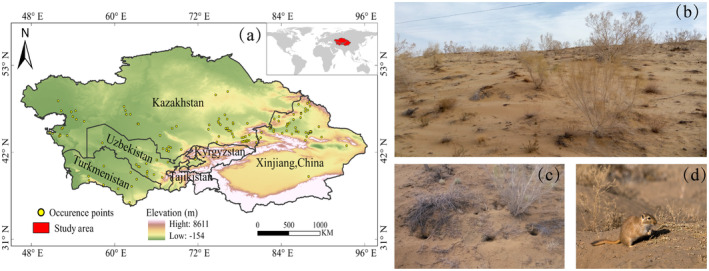
(a) Occurrence points of *Rhombomys opimus* population in Central Asia; (b) *Rhombomys opimus* habitat; (c) *Rhombomys opimus* burrows; and (d) *Rhombomys opimus* .

The study area defined in this research primarily includes Kazakhstan, Turkmenistan, Kyrgyzstan, Uzbekistan, Tajikistan, and China's Xinjiang (Chen et al. 2020) (Figure 1). These regions represent the principal habitat of *R. opimus* . The occurrence data of *R. opimus* were obtained through the following methods: (1) Field surveys conducted from 2016 to 2022 in regions such as Beishawo, Hoboksar Mongol Autonomous County, Manas County, and Fukang City in Xinjiang, China. The geographical coordinates and elevations of some *R. opimus* individuals were recorded using a global positioning system; these data are proprietary to our team. (2) The Global Biodiversity Information Facility (GBIF, https://www.gbif.org/, accessed on 10 August 2023). (3) Published papers with distribution data that included place names but lacked coordinates, for which we used the GPSSPG website (http://www.gpsspg.com) to obtain the coordinates. We collected 97, 249, and 20 occurrence records from these three sources. To mitigate spatial autocorrelation, we employed the “Spatially Sparse Occurrence Data” tool from the SDM toolbox (Brown, Bennett, and French 2017) to refine the distribution data of *R. opimus* within the study area so that only 1 occurrence point was retained in each 1 × 1 km raster. This process resulted in a final selection of 145 occurrence records (Figure 1).

### 2.2 Environment Variables

This study initially selected 56 variables potentially related to the habitat of *R. opimus* , including bioclimatic, topographic, soil, human footprint, and vegetation data (Table S1). The bioclimatic and elevation data were sourced from the WorldClim website (https://worldclim.org/) (Fick and Hijmans 2017). Soil data were obtained from the Harmonized World Soil Database v1.2 (HWSD) (Nachtergaele et al. 2012). ArcGIS software was used to extract data on slope and aspect. Vegetation growth in the study area was represented by the Normalized Difference Vegetation Index (NDVI). Human footprint data were acquired from the WCS Human Footprint dataset (https://wcshumanfootprint.org/) (Sanderson et al. 2022). For predictions under different climate scenarios, this study utilized historical bioclimatic data from 1970 to 2000 and future bioclimatic data from the ACCESS‐CM2 General Circulation Model (GCM) released by IPCC6. The ACCESS‐CM2 model provides more reliable simulations of annual precipitation patterns and long‐term precipitation trends in Central Asia (Guo et al. 2021). For predictions, this study utilized three Shared Socioeconomic Pathways (SSPs): SSP126, SSP245, and SSP585. Combined with the years 2030 (2020–2040) and 2050 (2040–2060), this results in six different scenarios: SSP126‐2030, SSP126‐2050, SSP245‐2030, SSP245‐2050, SSP585‐2030, and SSP585‐2050.

To address issues of autocorrelation and multicollinearity among variables, this study employed Spearman's correlation coefficient and the Jackknife method for variable selection (Worthington et al. 2016; Xu et al. 2024). Variables exhibiting correlation coefficients under 0.75 and those with higher contributions were chosen for modeling, resulting in a final selection of 16 variables (Table S3).

### 2.3 Habitat Distribution Modeling

In this study, we used the ENMeval (Warren et al. 2021) and biomod2 packages in R to analyze the suitable habitat distribution of *R. opimus* . First, we conducted MaxEnt model optimization using the ENMeval package in the R. This optimization focused on the two parameters provided by MaxEnt: feature classes (FCs) and the regularization multiplier (RM) (Phillips and Dudík 2008). The FCs include linear features (L), quadratic features (Q), hinge features (H), product features (P), and threshold features (T). The RM was set between 0.5 and 10, increasing by 0.5 with each run. We tested 120 parameter combinations using six FCs (L, LQ, H, LQH, LQHP, and LQHPT) and 20 RMs in the 0.5–10 range. The Akaike information criterion (AICc) was employed for assessment model complexity and fit, while the difference between training and testing AUC (Auc.diff.avg) and the 10% test omission rate (or10pct) were used to assess the model's performance on species occurrence points. The optimal model parameters with the lowest AICc value (delta.AICc = 0) were selected for modeling (Shi et al. 2023; Zhao et al. 2021). We then modeled the optimized MaxEnt and the other eight models, finally integrating the results using the biomod2 package in R. The predicted suitability results are continuous raster data representing the habitat suitability probability for *R. opimus* . These results need to be classified. First, we use the cutoff value derived from the receiver operating characteristic (ROC) curve to distinguish suitable from unsuitable habitats. The cutoff value, indicating the optimal threshold, is commonly used to assess habitat suitability (Liu et al. 2005). Based on expert experience and practical considerations, we further categorize the suitability results into four classes: unsuitable (< Cutoff), low suitability (Cutoff‐0.4), medium suitability (0.4–0.6), and high suitability (0.6–1) (Obunga et al. 2022).

### 2.4 Evaluation and Validation of the Model

In this research, both AUC and True Skill Statistic (TSS) metrics were employed to assess the predictive efficacy of the models. The area under the ROC curve, known as the area under the curve (AUC) value, is commonly used to assess the excellence of model predictions. It is not influenced by the occurrence rate of distribution points or the judgment threshold, making it a crucial method for the assessment of SDMs (Webb and Ting 2005). The TSS measures a model's ability to distinguish between “yes” and “no” predictions. It does not depend on the occurrence rate of distribution points but is influenced by the threshold. TSS is a straightforward metric for assessing the efficacy of SDMs (Farashi and Alizadeh‐Noughani 2023).

### 2.5 Distributional Changes and Migration Paths

We estimated the contemporary and forthcoming distribution regions of *R. opimus* and calculated their habitat suitability probabilities. Using ArcGIS, we identified the centroid of suitable habitats for each period and used the MCR model to delineate their movement routes. The MCR model can synthesize the intrinsic connections of ecological processes and reflect potential trends in species movement, allowing for the assessment of connectivity between target and source units. It finds extensive application in the design of ecological corridors (Dai, Liu, and Luo 2021; Wu et al. 2019). The formula is as follows:

(1) $$\mathrm{MCR}=f\;\min\;\sum_{j=n}^{i=m}D_{\mathrm{ij}}\times R_{i}$$

MCR stands for the minimum cumulative resistance, *f* is a monotonically increasing function, *D*_*ij*_ denotes the distance from source *j* to landscape unit *i*; and *R*_*i*_ represents the resistance coefficient of landscape unit *i* to species movement. This model necessitates data on sources and sinks. In this study, the current habitat centroid is used as the origin, and future habitat centroids serve as sinks across different climate scenarios. The formula for calculating the resistance surface (Keeley, Beier, and Gagnon 2016) is as follows:

(2) $$\left\{\begin{array}{cc} \text{resistance}=1 & \mathrm{HSI}\geq \text{threshold}\\ \text{resistence}=e^{\frac{\ln\;\left(0.001\right)}{\text{threshold}}\times \mathrm{HSI}}\times 1000 & \mathrm{HSI}<\text{threshold} \end{array}\right.$$

In the equation, the *threshold* is set to 0.6, and HSI represents the predicted habitat suitability index for *R. opimus* . According to the formula of the resistance surface, the final obtained resistance surface takes the range of 1–639 (Keeley, Beier, and Gagnon 2016). Additionally, we incorporated a land cover resistance layer. We overlaid the habitat suitability classification map with land cover types. Built‐up areas, permanent snow and ice, and water bodies were considered impassable obstacles and assigned a value of 639. Other non‐suitable land cover types were given a value of 14, reflecting the threshold (HIS = Cutoff = 0.3715) between suitable and unsuitable habitats. All other land cover types were assigned a resistance value of 1.

## 3 Results

### 3.1 Evaluation of Model Prediction Performance

With MaxEnt's default parameters (RM = 1, FC = LQHPT), forecasting the possible distribution of *R. opimus* resulted in a delta.AICc of 351.75. When RM = 5.5 and FC = LQHP, the delta.AICc was 0, and both Auc.diff.avg. and Or.10p.avg. were 43.4% and 33.3% lower, respectively, than those of the default model. According to the minimum AICc criterion, this model is considered optimal. This study ultimately selected RM = 5.5 and FC = LQHP as the final parameter settings for the MaxEnt model. AUC and TSS values were used to jointly evaluate the predictive performance of each model (Figure S1). The RF, GBM, and MaxEnt models ranked in the top three for both AUC and TSS among all algorithms (Figure S2).

**FIGURE 2:**
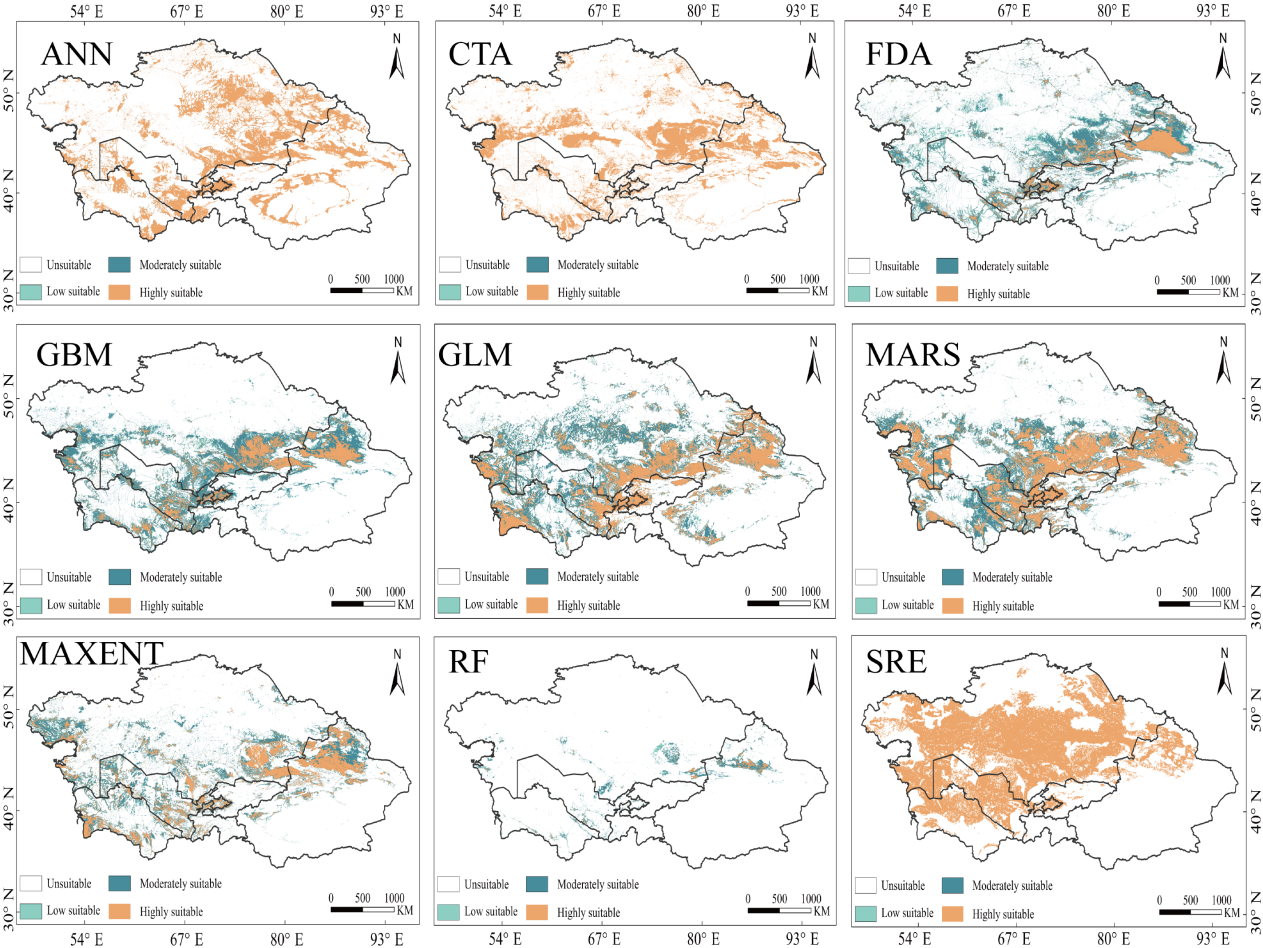
The prediction results of each model on *Rhombomys opimus* .

The nine algorithms used in this study can be categorized as regression algorithms (GLM and MARS), classification algorithms (FDA and CTA), machine learning algorithms (RF, GBM, ANN, and MaxEnt), and envelope algorithms (SRE). Regarding the spatial distribution predicted by the models, the regression algorithms, envelope algorithms, the CTA classification algorithm, and the ANN machine learning algorithm all overestimated the area of highly suitable habitats. Among the classification models, the traditional classification tree algorithm (CTA) and the envelope algorithm (SRE) performed the worst in spatial simulation (Figure 2). Both predicted the entire suitable habitat area as highly suitable, with contiguous distribution, providing no distribution details. They failed to show a smooth transition from low to high suitability areas, which does not align with the ecological traits of the species. With increasing model complexity, the FDA, GBM, MaxEnt, and RF models showed relatively better spatial performance. The highly suitable habitats generally covered the areas with sampling points without being overly extensive, and they provided some habitat distribution details. However, the FDA model overestimated the aggregate extent of suitable habitats. In terms of algorithm complexity, simpler models such as GLM, CTA, MARS, SRE, and FDA performed poorly, overestimating the distribution area. In contrast, more complex machine learning algorithms like GBM, RF, and MaxEnt showed more convergent spatial simulations, provided habitat distribution details, and exhibited better spatial performance.

The top three models, RF, GBM, and MaxEnt, were integrated into the ensemble model. The AUC score for the ensemble model was 0.986, and the TSS score was 0.899, indicating excellent predictive performance and reliability.

### 3.2 Model Predictions for *Rhombomys opimus* Under Current Climate Scenarios

**FIGURE 3:**
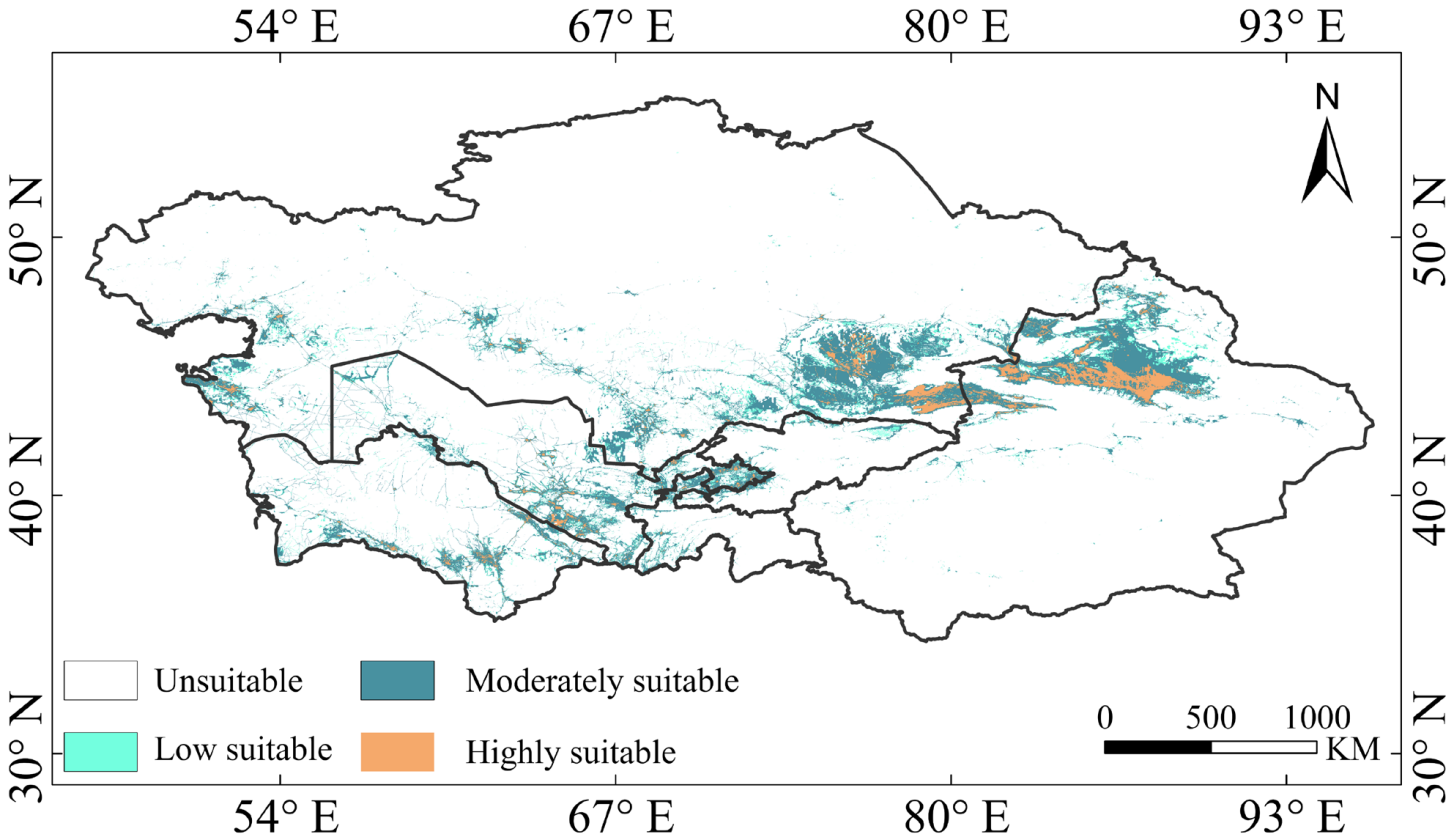
Distribution of the suitable habitat for *Rhombomys opimus* under current climate models.

Under the current climate model, the total suitable habitat area for *R. opimus* in the arid regions of Central Asia is 81.34 × 10^4^ km^2^, primarily concentrated in northern Xinjiang, southern Kazakhstan, and southeastern Uzbekistan (Figure 3). In particular, the region of high suitability is 9.99 × 10^4^ km^2^, mainly located in northern Xinjiang and southeastern Kazakhstan. The medium suitability area is 55.94 × 10^4^ km^2^, primarily in northern Xinjiang, southeastern Kazakhstan, southeastern Uzbekistan, and southern Turkmenistan. The low suitability area is 15.41 × 10^4^ km^2^, distributed along the edges of the medium suitability area and along the border regions of southern Kazakhstan, Kyrgyzstan, and the intersection of Uzbekistan and Tajikistan (Figure 3).

Based on the ensemble model predictions, the primary environmental factors (Table S2) impacting the potential geographic range of *R. opimus* are bio1 (24.56%), hfp (23.93%), bio17 (15.53%), bio19 (5.84%), ndvi (5.35%), elevation (3.83%), aspect (3.13%), bio9 (1.95%), and s‐gravel (1.52%). Their cumulative contribution rate is 85.63%.

### 3.3 Geographical Distribution of *Rhombomys opimus* Across Various Climate Scenarios

**FIGURE 4:**
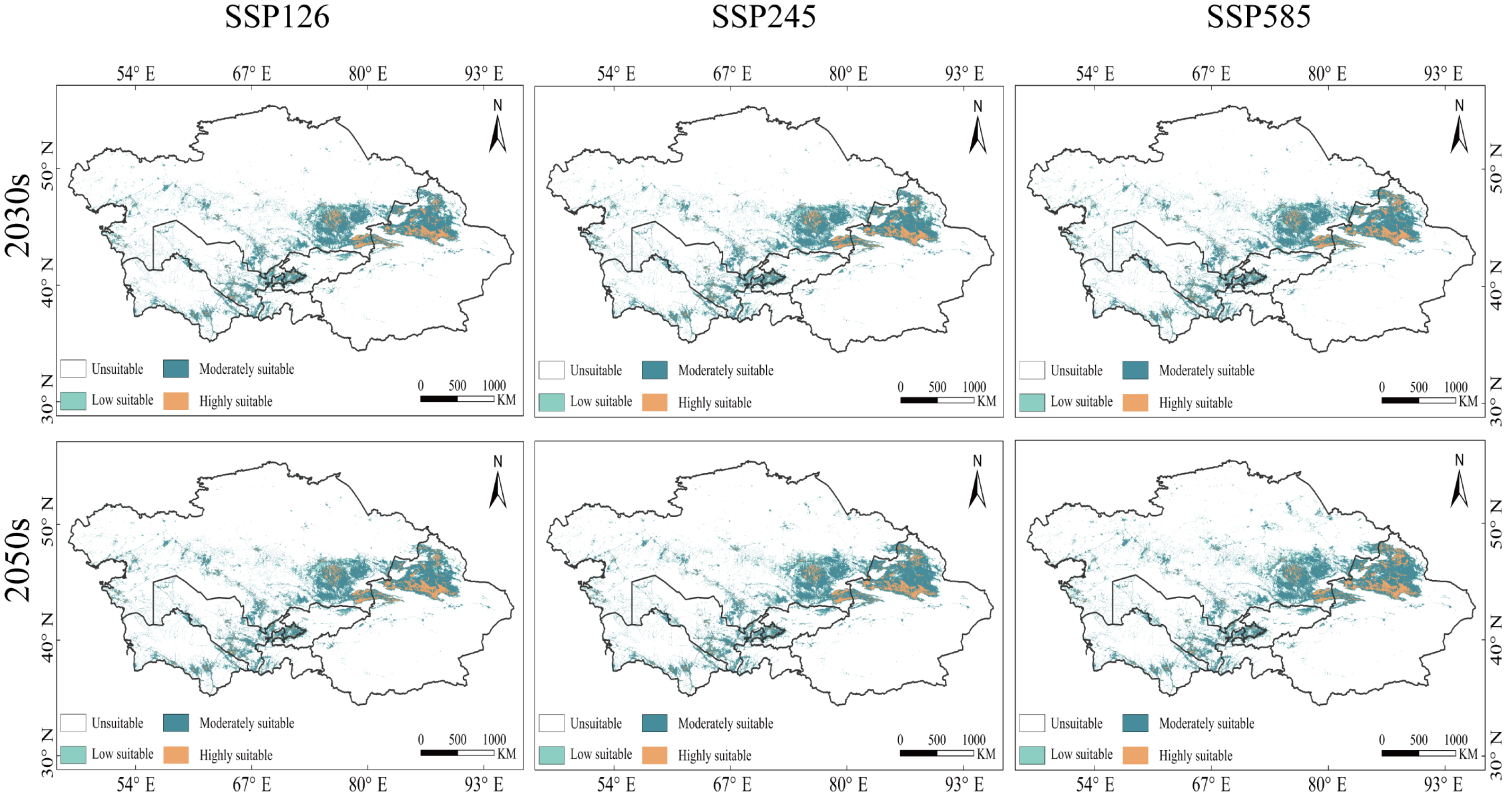
Projected potential habitats of *Rhombomys opimus* under future climate scenarios.

**FIGURE 5:**
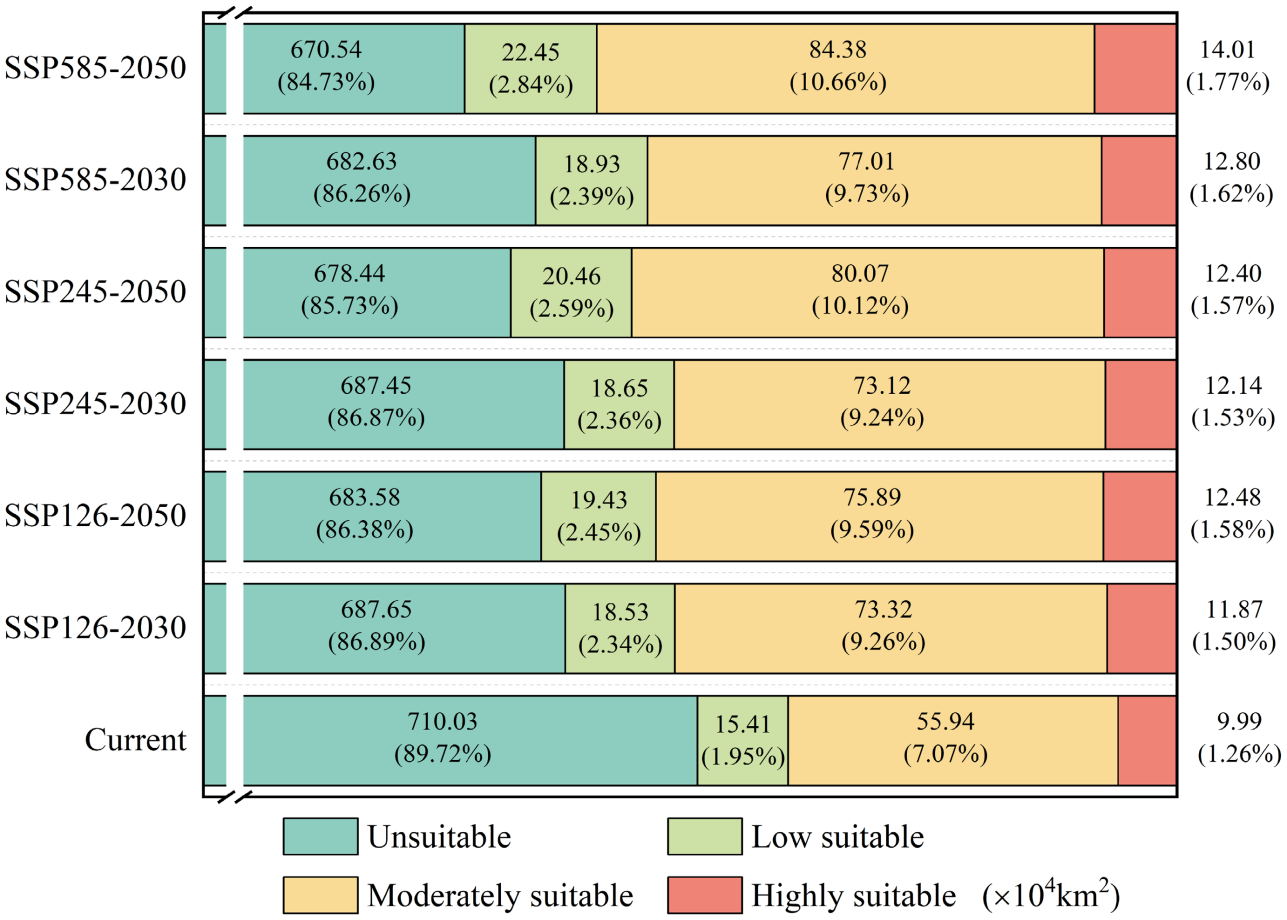
Habitat area and proportion of *Rhombomys opimus* under different climate change scenarios.

In the context of future climate scenarios, the predicted suitable habitat area for *R. opimus* ranges from 103.72 × 10^4^ km^2^ to 120.84 × 10^4^ km^2^, an increase of 2.83% to 4.99% compared to the current suitable area. The primary regions of distribution include northern Xinjiang in China, most of Almaty Province in Kazakhstan, as well as western Kashkadarya in Uzbekistan (Figure 4). Across various climate scenarios, the predicted area of elevated suitability levels for *R. opimus* ranges from 11.87 × 10^4^ km^2^ to 14.01 × 10^4^ km^2^, accounting for 1.50% to 1.77% (Figure 5), primarily in northern Xinjiang, China, and northern and eastern Almaty, Kazakhstan. The area of medium suitability ranges from 73.12 × 10^4^ km^2^ to 84.38 × 10^4^ km^2^, accounting for 9.24% to 10.66%, mainly in northern Xinjiang, eastern Almaty, and southeastern Uzbekistan. The area of low suitability habitats ranges from 18.53 to 22.45 × 10^4^ km^2^, accounting for 2.34% to 2.84% of the total, and is primarily distributed in northern Xinjiang, China, central Almaty, Kazakhstan, and northern Andijan, Uzbekistan. Among the different suitability levels, the medium suitability area shows the largest increase, while the low and high suitability areas show smaller increases.

Comparing the forecasted suitable habitat areas for different years under the same scenario, we discovered that the suitable habitat area for *R. opimus* in 2050 is larger than that in 2030 under all three climate scenarios (SSP126, SSP245, and SSP585). Specifically, the area increased by 0.51% from 2030 to 2050 under SSP126, by 1.14% under SSP245, and by 1.53% under SSP585. Additionally, as CO_2_ emission concentrations increase, the suitable habitat area for *R. opimus* shows an upward trend. Specifically, in 2030, the suitable habitat areas under SSP126, SSP245, and SSP585 are 103.72 × 10^4^ km^2^, 103.91 × 10^4^ km^2^, and 108.74 × 10^4^ km^2^, respectively. By 2050, these areas increase to 107.8 × 10^4^ km^2^, 112.93 × 10^4^ km^2^, and 120.84 × 10^4^ km^2^ under SSP126, SSP245, and SSP585, respectively.

### 3.4 Spatial Changes and Migration Routes of *Rhombomys opimus* Suitable Habitats

**FIGURE 6:**
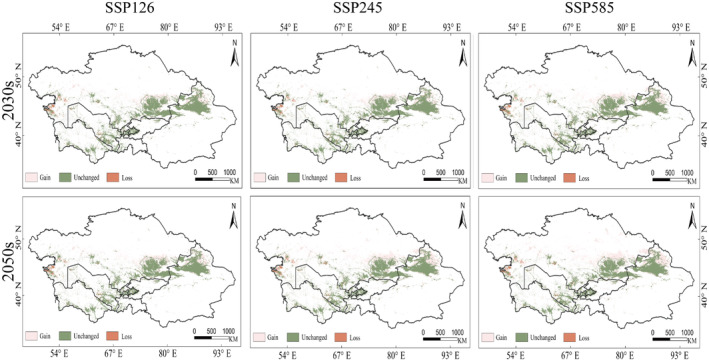
Spatial changes in the potential habitat of *Rhombomys opimus* under different future climate scenarios.

Analyzing the spatial changes between the present and future climate conditions for the suitable habitat of *R. opimus*, the results indicate that the expansion of suitable habitat areas under different SSPs is in the order of SSP126 < SSP245 < SSP585. This indicates that the expansion area of suitable habitats increases with higher CO_2_ concentrations. Under the SSP585‐2050 scenario (Figure 6), the expansion reaches 43.84 × 10^4^ km^2^ (Table 1), the largest among all scenarios, mainly expanding in northern Xinjiang, China; northern Almaty, Kazakhstan; southern Karaganda; and eastern Kazakhstan. Under the SSP245‐2030 scenario, the expansion area is 26.24 × 10^4^ km^2^, the smallest increase among all scenarios, mainly expanding in northern Xinjiang, China; northern Almaty; and southern Karaganda, Kazakhstan. Northern Xinjiang, China; northern Almaty; and southern Karaganda show a consistent expansion trend across all scenarios. The reduction in suitable habitat area follows the order SSP126 < SSP585 < SSP245, with the reduction area initially increasing and then decreasing with higher CO_2_ concentrations (Figure 6). Under the SSP245‐2050 scenario, the habitat area of *R. opimus* decreases the most, reaching 4.77 × 10^4^ km^2^, primarily in western Mangystau and southern Atyrau, Kazakhstan. The smallest reduction occurs under the SSP126‐2050 scenario, with a size of 3.55 × 10^4^ km^2^, mainly in western Mangystau. The area of unchanged regions varies as SSP245 < SSP585 < SSP126, with the area initially decreasing and then increasing with higher CO_2_ concentrations. Under the SSP126‐2050 scenario, the unchanged area of *R. opimus* is the largest, at 77.79 × 10^4^ km^2^, mainly in northern Xinjiang, China, and the Almaty region of Kazakhstan. Under the SSP245‐2050 scenario, the unchanged area is the smallest, at 76.57 × 10^4^ km^2^, primarily in northern Xinjiang, China, and the Almaty region of Kazakhstan.

**TABLE 1:** Alterations in the extent of suitable habitat of *Rhombomys opimus* under diverse climatic circumstances (×10^4^ km^2^).

Scenario	Gain		Loss		Unchanged	
SSP126‐2030	26.27	56.27	3.89	7.44	77.45	155.24
SSP126‐2050	30.00		3.55		77.79	
SSP245‐2030	26.24	62.6	3.66	8.43	77.68	154.25
SSP245‐2050	36.36		4.77		76.57	
SSP585‐2030	31.38	75.22	3.97	8.32	77.37	154.36
SSP585‐2050	43.84		4.35		76.99	

**FIGURE 7:**
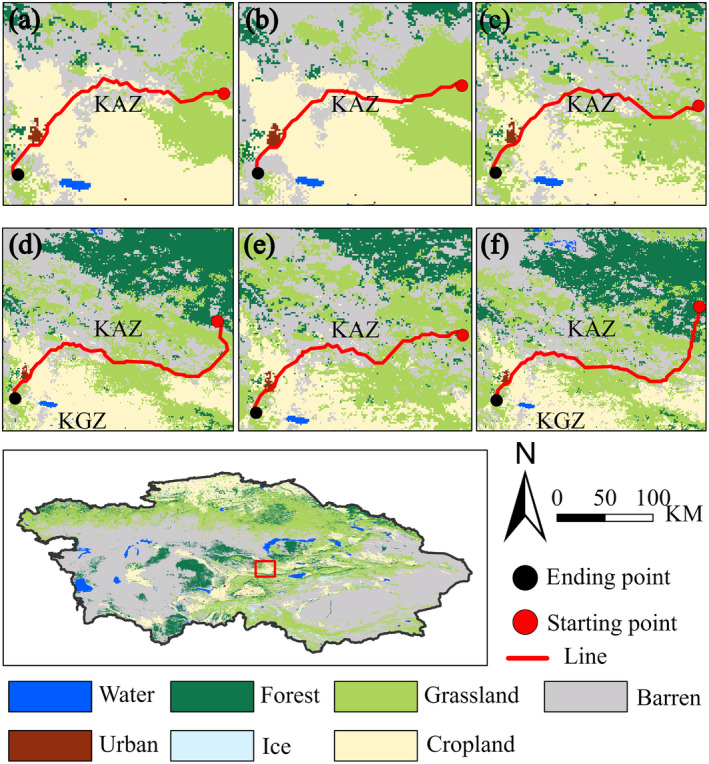
Comparison of the migration pathways of *Rhombomys opimus* under different land use types in the future. (a) SSP126‐2030; (b) SSP126‐2050; (c) SSP245‐2030; (d) SSP245‐2050; (e)SSP585‐2030; and (f) SSP585‐2050.

Using the MCR model, we calculated the migration routes of the suitable habitat centroids under the three SSPs in the 2030s and 2050s compared to the current centroids. The results show that under the current climate, the distribution center of *R. opimus* is located at the border between southwestern Zhambyl and Almaty in Kazakhstan, with coordinates 43.42° N, 73.67° E, and an elevation of 557 m. Across various climate scenarios, the distribution center of *R. opimus* shows a northward migration trend longitudinally and a shift to higher elevations vertically (Figure 7). Under the SSP126 climate scenario, the distribution center in the 2030s migrates 93.29 km northeast horizontally and moves 485 m to higher elevations vertically. The passing landscape is in the order of grassland—farmland—grassland. In the 2050s, the distribution center migrates 104.88 km northeast horizontally and moves 924 m to higher elevations vertically. The passing landscape is in the order of bare land—farmland—grassland. Under the SSP245 climate scenario, the distribution center in the 2030s migrates 96.14 km northeast horizontally and moves 496 m to higher elevations vertically. The passing landscape is in the order of grassland—farmland—bare land—bare land and grassland interspersed zones. In the 2050s, the distribution center migrates 156.27 km northeast horizontally and moves 113 m to higher elevations vertically. The passing landscape is in the order of grassland—farmland—grassland—forest. Under the SSP585 climate scenario, the distribution center in the 2030s migrates 126.82 km northeast horizontally and moves 340 m to higher elevations vertically. The passing landscape is in the order of grassland—farmland—bare land—grassland. In the 2050s, the distribution center migrates 181.05 km northeast horizontally and moves 136 m to higher elevations vertically. The passing landscape is in the order of grassland—farmland—bare land—forest.

## 4 Discussion

### 4.1 Comparative Analysis of Integrated SDMs and Single SDMs

The study results indicate that, for the same input data, complex machine learning models exhibit better statistical accuracy and superior spatial performance compared to other classification, regression, and envelope models. Firstly, regression models output continuous values from 0 to 1 (Kasza and Wolfe 2014), allowing them to express varying habitat suitability levels better than classification and envelope models. However, regression models respond poorly to complex variables (Burks, Randolph, and Seida 2019), and their predictions primarily fit the basic ecological niche of the species. This results in an idealized distribution of suitable habitats that only occurs under highly favorable environmental conditions with abundant resources, leading to an overestimation of highly suitable areas. On the other hand, classification and envelope models can only differentiate between presence and absence, failing to capture the variations within each category (Lee and Su 2020; Scholz and Wimmer 2021). This limitation makes them less effective for grading suitability levels. Although machine learning models perform well, they have poor portability, lack clear statistical principles, and are prone to overfitting (Montesinos López, Montesinos López, and Crossa 2022). The predictions often exhibit spatial distribution uncertainties, even when the model's statistical accuracy is high. This is particularly true for ANN models, which have complex algorithms and significant randomness in modeling, leading to high variability in results across multiple runs (Liu and Wang 2021).

Even when using the same dataset, different models produce varying results, leading to unavoidable uncertainties in spatial performance. A single model's spatial performance may not accurately represent the true distribution of the species. Additionally, we typically use models based solely on the given dataset. When spatiotemporal conditions change, the same model cannot be used for accurate modeling (Valavi et al. 2022). Adopting an ensemble modeling strategy addresses this issue. Some researchers have integrated the best‐performing GBM and RF models for *R. opimus* (Wen et al. 2022), consistent with this study's findings. However, they did not optimize the MaxEnt model, using only default settings, resulting in suboptimal performance and exclusion from their ensemble. In this research, optimization of the MaxEnt model was conducted, achieving AUC and TSS values comparable to the GBM model. The final ensemble strategy for *R. opimus* included RF, GBM, and MaxEnt models. Because the ensemble model maps the main trends (e.g., mean, median, or other percentiles) and overall variations (including uncertainties) of all models (Forester, DeChaine, and Bunn 2013), it provides a valuable reference for future ensemble modeling strategies.

### 4.2 Geographical Distribution of *Rhombomys opimus*

As per the model predictions, the primary variables influencing the potential geographic range of *R. opimus* are annual mean temperature (bio1), human footprint (hfp), precipitation of the driest quarter (bio17), precipitation of the coldest quarter (bio19), and NDVI. Under the current climate model, *R. opimus* is primarily distributed in central and northern Xinjiang, China, southern Kazakhstan, and eastern Uzbekistan. Food availability, terrain, and vegetation cover within the habitat are key factors influencing the species' habitat selection. For *R. opimus*, desert plants are the most important food source, and their population changes directly depend on the condition of these plants. The abundance of vegetation in deserts is primarily determined by precipitation from October to May of the following year (Du et al. 2017; Wang et al. 2020). The model also indicates that precipitation during the coldest season (bio19) from October to December and the driest season (bio17) from November to April significantly affects the dispersion of suitable habitats for *R. opimus*. In years with higher precipitation during these periods, the population of *R. opimus* increases, while in years with lower precipitation, their population decreases. A warm spring favors the breeding of *R. opimus*. During the extremely unfavorable winter months in terms of climate and food conditions, the mortality rate of *R. opimus* is very high. A comparably gentle winter helps in the recovery of their population. Conversely, prolonged hot summers are detrimental to the development of desert vegetation (Zhang et al. 2018), thus negatively impacting the survival of *R. opimus*. Therefore, a relatively stable annual mean temperature is crucial for their survival.

Researchers from the Xinjiang Institute of Desert Ecology in China have divided the arid regions of Central Asia into three climatic zones (Dilinuer et al. 2021): temperate continental climate, arid desert climate, and quasi‐Mediterranean climate. The arid desert climate zone, ranging approximately from 36° N to 51° N and 48° E to 80° E, primarily includes southwestern Kazakhstan and parts of Uzbekistan and Turkmenistan and encompasses the pair of most expansive deserts, the Karakum and Kyzylkum deserts. This closely aligns with the model‐predicted suitable habitat range for *R. opimus* under the current climate model. This alignment is due to the arid desert climate zone being mainly influenced by low‐latitude circulation systems (Song et al. 2023), with precipitation concentrated in the winter and spring, meeting *R. opimus*'s requirements for cold‐season and dry‐season precipitation. Additionally, in Xinjiang, winter and spring precipitation is highest in the western Tianshan, western Northern Xinjiang, and northern regions (Guan, Yao, and Schneider 2022; Yue, Xu, and Wang 2022), further confirming that *R. opimus* prefers areas with significant precipitation from October to April.

### 4.3 Migratory Pathways in the Center of the *Rhombomys opimus* 's Fitness Zone

The findings of this study reveal that under all future scenarios, the suitable habitat of *R. opimus* exhibits an overall expansion trend and a shift toward higher latitudes and elevations. The expansion area and migration distance are positively correlated with CO_2_ emission concentrations and time duration. With global warming, more areas on land will face significant increases or decreases in precipitation. Some researchers point out that the high‐altitude regions of eastern and southern Central Asia will experience the most significant increase in future precipitation, particularly under high‐emission scenarios (Yao et al. 2021). Alterations in the frequency or intensity of rainfall events may alter desert plant and animal communities. Higher atmospheric CO_2_ concentrations and more intense rainfall events will increase opportunities for plant growth in deserts. Elevated CO_2_ and increased precipitation greatly promote the growth of C4 plants. (Nie et al. 2018). *Haloxylon ammodendron*, a typical C4 plant and a primary food source for *R. opimus* (Qiang et al. 2023; Wen et al. 2020), will benefit from increased precipitation in eastern Central Asia, leading to a certain expansion of *R. opimus* suitable habitats.

As temperatures rise and environmental conditions change, many species face habitat loss and fragmentation (Holyoak and Heath 2016), disrupting the delicate balance between species. This disruption causes cascading effects across trophic levels, leading to profound changes in ecosystems. The consequences are particularly evident for animals. Higher temperatures cause vegetation zones to migrate toward higher latitudes and elevations (Kubelka et al. 2022), disrupting herbivores' access to specific plant species. This mismatch in food supply affects the entire food chain (Tylianakis et al. 2008). Under the backdrop of climate change, the habitat environment of *R. opimus* will also undergo profound changes. During periods of warming, land temperature increases will significantly raise the potential evaporation in Central Asia, making the region hotter and drier. Over time, from 2015 to 2100, the global dry‐wet variability rate is expected to decline, with Central Asia facing a higher risk of intensified drought (Zhou et al. 2023). As a rodent species with strict habitat requirements, *R. opimus* often engages in short‐distance dispersal due to food scarcity. In the coming decades, *R. opimus* will respond to changes in habitat and food availability, potentially undertaking long‐distance dispersal or migration (Wen et al. 2022) during periods of extreme food scarcity. Under the same SSP scenario, the migration distance of *R. opimus* increases over time, indicating that as drought events intensify in Central Asia, their migration behavior becomes more frequent. Researchers believe that the trend of increasing drought risk is more severe under higher SSP emission scenarios (Su et al. 2021) and when comparing greater gerbils in the same year under different SSP emission scenarios. Comparing the migration distances of *R. opimus* under different SSP scenarios for the same year, it can be seen that higher emission scenarios result in longer migration distances. Notably, under the SSP585‐2050 scenario, *R. opimus* exhibits unprecedented large‐scale migration.

Additionally, this study found that the migration routes of *R. opimus* primarily traverse farmland, grassland, and bare land. Desert forest and grassland areas are the main habitats for *R. opimus* and are their preferred migration routes. Due to climate change, food supply along these migration routes is unstable. Farmland, with stable vegetation growth due to human intervention, provides a reliable and steady food source for *R. opimus* during migration when food is scarce. Thus, during migration, *R. opimus* prefers to navigate the edges of farmland and bare land or grassland and bare land (Figure 7). This poses a significant threat to food security and grassland productivity. While climate and food factors are significant influences on *R. opimus* migration, competition for resources within and between species and changes in predator–prey distributions are also important reasons for migration.

### 4.4 Policy Recommendations

Studies have found that the suitable habitat of *R. opimus* shows an overall expansion trend in the future. To mitigate and address this change, the subsequent recommendations are suggested based on the study's findings:

For the prevention of *R. opimus* damage, reducing carbon emissions is an effective measure since the expansion of suitable habitats is significantly less under low‐emission scenarios compared to high‐emission scenarios. In terms of management, northern Xinjiang in China and southern Kazakhstan will be the main expansion areas of *R. opimus* suitable habitats under future climate change. Particularly, a belt of suitable habitat expansion will form in northern Almaty Province, Kazakhstan—Tacheng Region, Xinjiang, China—Altay Region, and Xinjiang, China. Xinjiang is likely to become a major destination for *R. opimus* migration, necessitating strengthened cooperation between China and Kazakhstan in rodent control and focused prevention in these areas, with a scientific allocation of control resources. Control strategies must consider the interconnectedness of ecosystems and emphasize biodiversity conservation. Extermination measures are not advisable; instead, more reasonable biological control methods should be employed. To mitigate the damage caused by *R. opimus*, it is important to note that when selecting migration routes, they prioritize grasslands as food supply stations. However, in the absence of grasslands, farmlands become the primary choice, posing significant risks to human health and food production safety. Therefore, future farmland expansion efforts should make appropriate concessions for grassland conservation.

### 4.5 Limitations and Prospects

Future climate change contains many uncertainties. This study only selected the ACCESS‐CM2 global circulation model to predict the potential suitable habitats for *R. opimus*, which may increase the uncertainty of SDM predictions, leading to discrepancies in the anticipated geographic distribution and occurrence range of *R. opimus*. This, in turn, can impact policymakers' ability to develop effective ecological protection measures. Some researchers use several GCMs for modeling predictions, which significantly minimize uncertainty and inaccuracies (Jinga, Liao, and Nobis 2021; Thuiller et al. 2019). Although the migration routes and suitable habitat changes of *R. opimus* are influenced by climate, terrain, food availability, and land cover types, population competition and predator distribution are also important factors affecting migration routes and habitat changes. In the future, these factors should be included in the development of migration routes and SDMs.

## 5 Conclusion

This study adopted an ensemble modeling approach to investigate changes in the dispersion and migration routes of *R. opimus* suitable habitats in the arid regions of Central Asia under future climate variability scenarios. The results indicate:

1. For forecasting the potential spatial distribution of *R. opimus*, the integrated model of RF, GBM, and MaxEnt exhibits excellent performance in both statistical accuracy and spatial distribution simulation.
2. Climate change will facilitate the expansion of suitable habitats for *R. opimus*, primarily in northern Xinjiang, China, and northern Almaty, Kazakhstan. The extent of this expansion is positively correlated with CO₂ emission concentrations and is projected to increase over time.
3. Under different SSPs, the suitable habitats of *R. opimus* exhibit a tendency to shift toward higher altitudes and latitudes. In terms of migration strategy, the species shows a preference for moving through grasslands and farmlands areas.

## Author Contributions

**Xuan Liu:** data curation (equal), formal analysis (equal), investigation (equal), methodology (equal), software (equal), visualization (equal), writing – original draft (equal). **Li Xu:** software (equal), validation (equal), writing – review and editing (equal). **Jianghua Zheng:** conceptualization (equal), funding acquisition (equal), resources (equal), supervision (equal), writing – review and editing (equal). **Jun Lin:** investigation (equal), project administration (equal), writing – review and editing (equal). **Xuan Li:** investigation (equal), project administration (equal), writing – review and editing (equal). **Liang Liu:** formal analysis (equal), visualization (equal), writing – review and editing (equal). **Ruikang Tian:** investigation (equal), writing – review and editing (equal). **Chen Mu:** writing – review and editing (equal).

## Conflicts of Interest

The authors declare no conflicts of interest.

## Supporting information


Data S1:

## Data Availability

The data and materials underlying this article are available in the article and in its Supporting Information.
