# Supplementary material for: Great Gerbils (Rhombomys opimus) in Central Asia Are Spreading to Higher Latitudes and Altitudes
Source: Ecol Evol. 2024 Nov 11;14(11):e70517. doi: 10.1002/ece3.70517 (PMC11554374; doi:10.1002/ece3.70517)
Supplement: Supplementary file 1 — Data S1: [file ECE3-14-e70517-s001.docx]

**Supplementary Materials**

**Table. S1.** **Initially selected environment variables**

| Variables | Description | Unit |
| --- | --- | --- |
| bio1 | Annual Mean Temperature | ℃ |
| bio2 | Mean Diurnal Range | ℃ |
| bio3 | Isothermally | % |
| bio4 | Temperature Seasonality | Standard Deviation ×100 |
| bio5 | Max Temperature of Warmest Month | ℃ |
| bio6 | Min Temperature of Coldest Month | ℃ |
| bio7 | Annual Range of Temperature | ℃ |
| bio8 | Mean Temperature of Wettest Quarter | ℃ |
| bio9 | Mean Temperature of Driest Quarter | ℃ |
| bio10 | Mean Temperature of Warmest Quarter | ℃ |
| bio11 | Mean Temperature of Coldest Quarter | ℃ |
| bio12 | Annual Precipitation | mm |
| bio13 | Precipitation of Wettest Month | mm |
| bio14 | Precipitation of Driest Month | mm |
| bio15 | Precipitation Seasonality | Coefficient of Variation |
| bio16 | Precipitation of Wettest Quarter | mm |
| bio17 | Precipitation of Driest Quarter | mm |
| bio18 | Mean Precipitation of Warmest Quarter | mm |
| bio19 | Mean Precipitation of Coldest Quarter | mm |
| t_gravel | Topsoil Gravel Content | %vol. |
| t_sand | Topsoil Sand Fraction | % wt. |
| t_silt | Topsoil Silt Fraction | % wt. |
| t_caly | Topsoil Clay Fraction | % wt. |
| t_usda_tex_class | Topsoil USDA Texture Classification | name |
| t_ref_bulk_density | Topsoil Reference Bulk Density | kg/dm3 |
| t_oc | Topsoil Organic Carbon | % weight |
| t_ph_h2o | Topsoil pH (H2O) | -log(H^+^) |
| t_cec_clay | Topsoil CEC (clay) | cmol/kg |
| t_cec_soil | Topsoil CEC (soil) | cmol/kg |
| t_bs | Topsoil Base Saturation | % |
| t_teb | Topsoil TEB | cmol/kg |
| t_caco3 | Topsoil Calcium Carbonate | % weight |
| t_caso4 | Topsoil Gypsum | % weight |
| t_esp | Topsoil Sodicity (ESP) | % |
| t_ece | Topsoil Sodicity (ESP) | dS/m |
| s_ref_bulk_density | Subsoil Reference Bulk Density | kg/dm3 |
| s_bs | Subsoil Base Saturation | % |
| s_caco3 | Subsoil Calcium Carbonate | % weight |
| s_caso4 | Subsoil Gypsum | % weight |
| s_cec_clay | Subsoil CEC (clay) | cmol/kg |
| s_cec_soil | Subsoil CEC (soil) | cmol/kg |
| s_clay | Subsoil Clay Fraction | % |
| s_ece | Subsoil Salinity (ECe) | dS/m |
| s_esp | Subsoil Sodicity (ESP) | % |
| s_gravel | Subsoil Gravel Content | % |
| s_oc | Subsoil Organic Carbon | % weight |
| s_ph_h2o | Subsoil pH (H2O) | -log(H+) |
| s_usda_tex_class | Subsoil USDA Texture Classification | name |
| s_sand | Subsoil Sand Fraction | % |
| s_silt | Subsoil Silt Fraction | % |
| s_teb | Subsoil TEB | cmol/kg |
| NDVI | Normalized Differnce Vegetation Index | — |
| altitude | Altitude | m |
| slope | Slope | ° |
| aspect | Aspect | ° |
| Hf | Human Footprint | — |

**Table. S2.** ***Rhombomys opimus*** **occurrence point data**

| species | | longitude | | latitude | |
| --- | --- | --- | --- | --- | --- |
| Rhombomys_opimus | | 62.166672 | | 35.63905 | |
| Rhombomys_opimus | | 89.391665 | | 45.222015 | |
| Rhombomys_opimus | | 82.34 | | 45.02 | |
| Rhombomys_opimus | | 82.48 | | 45.04 | |
| Rhombomys_opimus | | 82.34 | | 45.11 | |
| Rhombomys_opimus | | 86.12 | | 44.63 | |
| species | | longitude | | latitude | |
| Rhombomys_opimus | | 80.45 | | 43.98 | |
| Rhombomys_opimus | | 87.648621 | | 43.974888 | |
| Rhombomys_opimus | | 87.451102 | | 43.499062 | |
| Rhombomys_opimus | | 84.854028 | | 45.597247 | |
| Rhombomys_opimus | | 85.147559 | | 45.707748 | |
| Rhombomys_opimus | | 85.750106 | | 46.13233 | |
| Rhombomys_opimus | | 93.393689 | | 42.826689 | |
| Rhombomys_opimus | | 82.515138 | | 45.16039 | |
| Rhombomys_opimus | | 82.918908 | | 44.591448 | |
| Rhombomys_opimus | | 82.91123 | | 44.835076 | |
| Rhombomys_opimus | | 81.15386 | | 44.507688 | |
| Rhombomys_opimus | | 83.659893 | | 46.519731 | |
| Rhombomys_opimus | | 87.739955 | | 48.192983 | |
| Rhombomys_opimus | | 86.482021 | | 48.088513 | |
| Rhombomys_opimus | | 88.120357 | | 47.838393 | |
| Rhombomys_opimus | | 88.057185 | | 48.361153 | |
| Rhombomys_opimus | | 87.799007 | | 47.330794 | |
| Rhombomys_opimus | | 87.444698 | | 47.174194 | |
| Rhombomys_opimus | | 85.518408 | | 44.30437 | |
| Rhombomys_opimus | | 83.616504 | | 45.953371 | |
| Rhombomys_opimus | | 82.982974 | | 46.195574 | |
| Rhombomys_opimus | | 85.766913 | | 46.788022 | |
| Rhombomys_opimus | | 81.042044 | | 43.854059 | |
| Rhombomys_opimus | | 87.520674 | | 44.495584 | |
| Rhombomys_opimus | | 86.447885 | | 44.556569 | |
| Rhombomys_opimus | | 89.781072 | | 43.908726 | |
| Rhombomys_opimus | | 84.119035 | | 44.398479 | |
| species | | longitude | | latitude | |
| Rhombomys_opimus | | 84.768028 | | 44.699871 | |
| Rhombomys_opimus | | 86.032495 | | 45.102649 | |
| Rhombomys_opimus | | 87.477509 | | 47.516324 | |
| Rhombomys_opimus | | 61.1833 | | 36.3171 | |
| Rhombomys_opimus | | 51.864626 | | 48.514516 | |
| Rhombomys_opimus | | 53.168587 | | 44.09924 | |
| Rhombomys_opimus | | 51.078071 | | 44.410513 | |
| Rhombomys_opimus | | 75.02129 | | 46.236647 | |
| Rhombomys_opimus | | 78.555782 | | 43.882856 | |
| Rhombomys_opimus | | 78.838264 | | 44.003301 | |
| Rhombomys_opimus | | 78.569947 | | 43.385019 | |
| Rhombomys_opimus | | 79.012613 | | 43.477116 | |
| Rhombomys_opimus | | 52.016678 | | 44.023312 | |
| Rhombomys_opimus | | 52.133855 | | 44.235621 | |
| Rhombomys_opimus | | 63.327133 | | 45.598335 | |
| Rhombomys_opimus | | 79.341888 | | 43.484928 | |
| Rhombomys_opimus | | 63.4552 | | 45.577411 | |
| Rhombomys_opimus | | 78.85 | | 43.38 | |
| Rhombomys_opimus | | 76.197444 | | 46.202051 | |
| Rhombomys_opimus | | 79.097835 | | 45.651847 | |
| Rhombomys_opimus | | 77.054378 | | 45.574998 | |
| Rhombomys_opimus | | 75.867854 | | 46.731741 | |
| Rhombomys_opimus | | 77.032405 | | 45.728591 | |
| Rhombomys_opimus | | 80.38039 | | 43.227835 | |
| Rhombomys_opimus | | 79.018085 | | 42.745641 | |
| Rhombomys_opimus | | 74.711445 | | 43.291841 | |
| Rhombomys_opimus | | 75 | | 44 | |
| species | | longitude | | latitude | |
| Rhombomys_opimus | | 55.5967 | | 45.90204 | |
| Rhombomys_opimus | | 63.183333 | | 38.583333 | |
| Rhombomys_opimus | | 56.25 | | 38.96667 | |
| Rhombomys_opimus | | 62.325241 | | 35.27301 | |
| Rhombomys_opimus | | 56.285889 | | 38.436581 | |
| Rhombomys_opimus | | 79.06498 | | 43.355503 | |
| Rhombomys_opimus | | 71.616216 | | 41.090749 | |
| Rhombomys_opimus | | 64.669941 | | 39.315856 | |
| Rhombomys_opimus | | 88.284472 | | 44.537525 | |
| Rhombomys_opimus | | 88.284472 | | 44.627617 | |
| Rhombomys_opimus | | 88.158019 | | 44.537398 | |
| Rhombomys_opimus | | 88.158272 | | 44.627544 | |
| Rhombomys_opimus | | 58.39108807 | | 37.87399412 | |
| Rhombomys_opimus | | 87.88473669 | | 44.37483499 | |
| Rhombomys_opimus | | 63.1269836 | | 40.4303198 | |
| Rhombomys_opimus | | 84.779203 | | 45.292002 | |
| Rhombomys_opimus | | 78.31323828 | | 43.83344896 | |
| Rhombomys_opimus | | 68.28106425 | | 45.02625939 | |
| Rhombomys_opimus | | 84.94913176 | | 45.52388577 | |
| Rhombomys_opimus | | 50.686342 | | 44.476946 | |
| Rhombomys_opimus | | 54.38265125 | | 47.25226847 | |
| Rhombomys_opimus | | 53.75301 | | 47.105566 | |
| Rhombomys_opimus | | 63.593635 | | 40.14597167 | |
| Rhombomys_opimus | | 52.38268614 | | 45.00210541 | |
| Rhombomys_opimus | | 52.31010409 | | 47.26382452 | |
| Rhombomys_opimus | | 74.90938317 | | 45.88600681 | |
| Rhombomys_opimus | | 66.06610284 | | 41.03275442 | |
| species | | longitude | | latitude | |
| Rhombomys_opimus | | 52.5262585 | | 45.0780291 | |
| Rhombomys_opimus | | 61.67861505 | | 46.78826844 | |
| Rhombomys_opimus | | 62.27429676 | | 46.80559263 | |
| Rhombomys_opimus | | 61.5422752 | | 47.28951394 | |
| Rhombomys_opimus | | 79.37457221 | | 44.19298448 | |
| Rhombomys_opimus | | 79.03636424 | | 43.52557666 | |
| Rhombomys_opimus | | 68.90149291 | | 45.27913997 | |
| Rhombomys_opimus | | 75.23785247 | | 45.4817569 | |
| Rhombomys_opimus | | 63.42314585 | | 40.17282971 | |
| Rhombomys_opimus | | 67.79918366 | | 42.68801787 | |
| Rhombomys_opimus | | 66.96074139 | | 42.45498478 | |
| Rhombomys_opimus | | 67.4822302 | | 42.20977515 | |
| Rhombomys_opimus | | 54.47875167 | | 46.15951167 | |
| Rhombomys_opimus | | 53.98472 | | 46.51393667 | |
| Rhombomys_opimus | | 67.66555176 | | 40.75915574 | |
| Rhombomys_opimus | | 52.15131417 | | 44.09482345 | |
| Rhombomys_opimus | | 58.22058274 | | 43.18442335 | |
| Rhombomys_opimus | | 78.53222994 | | 43.33499019 | |
| Rhombomys_opimus | | 64.70274104 | | 39.57223963 | |
| Rhombomys_opimus | | 78.75180231 | | 43.41815323 | |
| Rhombomys_opimus | | 68.08717745 | | 42.57726613 | |
| Rhombomys_opimus | | 77.10769314 | | 43.94321907 | |
| Rhombomys_opimus | | 76.95164753 | | 44.31767862 | |
| Rhombomys_opimus | | 67.52384431 | | 42.44527829 | |
| Rhombomys_opimus | | 69.59010098 | | 42.34161858 | |
| Rhombomys_opimus | | 67.77428996 | | 42.11730911 | |
| Rhombomys_opimus | | 76.02446124 | | 44.94626627 | |
| species | | longitude | | latitude | |
| Rhombomys_opimus | | 64.68625698 | | 38.8839398 | |
| Rhombomys_opimus | | 61.34492021 | | 47.7871707 | |
| Rhombomys_opimus | | 64.57076448 | | 40.55411276 | |
| Rhombomys_opimus | | 73.505472 | | 44.885083 | |
| Rhombomys_opimus | | 73.61601299 | | 45.45889873 | |
| Rhombomys_opimus | | 78.326519 | | 43.326801 | |
| Rhombomys_opimus | | 58.438661 | | 40.252113 | |
| Rhombomys_opimus | | 66.54 | | 44.11 | |
| Rhombomys_opimus | | 86.763472 | | 46.392194 | |
| Rhombomys_opimus | | 86.13049 | | 44.72321 | |
| Rhombomys_opimus | | 86.83059 | | 46.94916 | |
| Rhombomys_opimus | | 86.82985 | | 46.87247 | |
| Rhombomys_opimus | | 87.857 | | 44.413 | |
| Rhombomys_opimus | | 88.16 | | 44.583 | |
| Rhombomys_opimus | | 87.917 | | 38.933 | |
| Rhombomys_opimus | | 76.5 | | 44.717 | |
| Rhombomys_opimus | | 76.75 | | 44.667 | |
| Rhombomys_opimus | | 76.333 | | 44.75 | |
| Rhombomys_opimus | | 76 | | 45 | |
| Rhombomys_opimus | | 75 | | 45.7 | |
| Rhombomys_opimus | | 76.25 | | 45.05 | |
| Rhombomys_opimus | | 62.3 | | 39.44 | |
| Rhombomys_opimus | | 60.5 | | 37.383 | |
| Rhombomys_opimus | | 65.667 | | 39.167 | |
| Rhombomys_opimus | | 62.45 | | 37.667 | |
| Rhombomys_opimus | | 74.55 | | 49.27 | |
| Rhombomys_opimus | | 76.28 | | 49.69 | |
| species | | longitude | | latitude | |
| Rhombomys_opimus | | 82.333 | | 45.5 | |
| Rhombomys_opimus | | 66.833 | | 38.833 | |
| Rhombomys_opimus | | 80.744 | | 43.578 | |
| Rhombomys_opimus | | 80.63 | | 43.681 | |

**Table. S3.** **Filtered environment variables used to build the model**

| Variables | Description | Unit |
| --- | --- | --- |
| bio1 | Annual Mean Temperature | ℃ |
| bio4 | Temperature Seasonality | Standard Deviation ×100 |
| bio9 | Mean Temperature of Driest Quarter | ℃ |
| bio17 | Precipitation of Driest Quarter | mm |
| bio19 | Mean Precipitation of Coldest Quarter | mm |
| t_caso4 | Topsoil Gypsum | % weight |
| t_esp | Topsoil Sodicity (ESP) | % |
| s_bs | Subsoil Base Saturation | % |
| s_caco3 | Subsoil Calcium Carbonate | % weight |
| s_cec_soil | Subsoil CEC (soil) | cmol/kg |
| s_gravel | Subsoil Gravel Content | % |
| s_oc | Subsoil Organic Carbon | % weight |
| NDVI | Normalized Differnce Vegetation Index | — |
| slope | Slope | ° |
| aspect | Aspect | ° |
| Hf | Human Footprint | — |

**
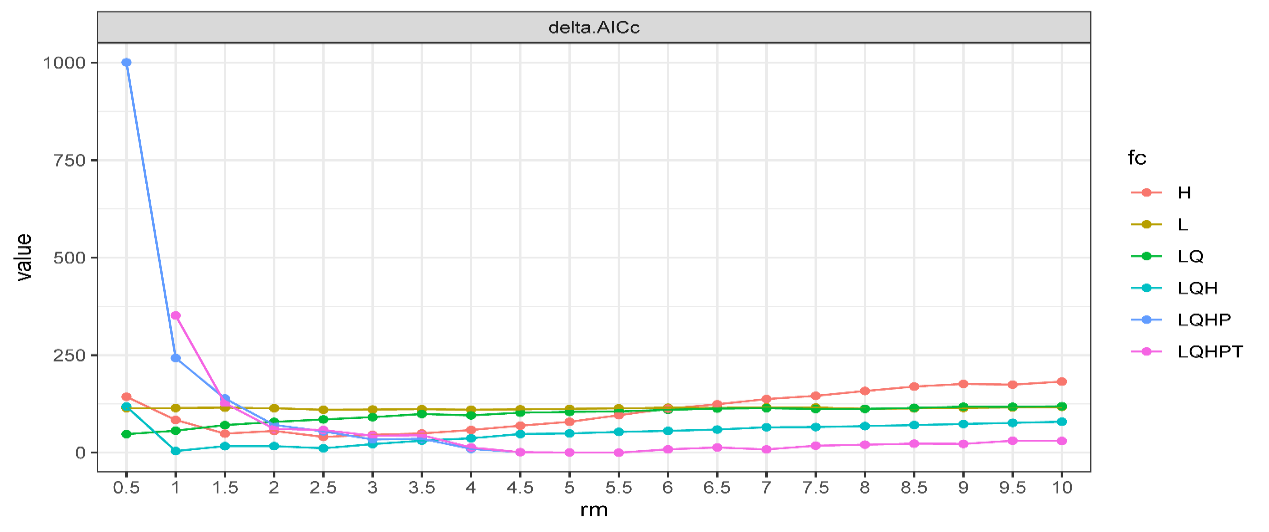
**

**Fig. S1.** **The delta. AICc values of MaxEnt model under different parameter combinations**

**
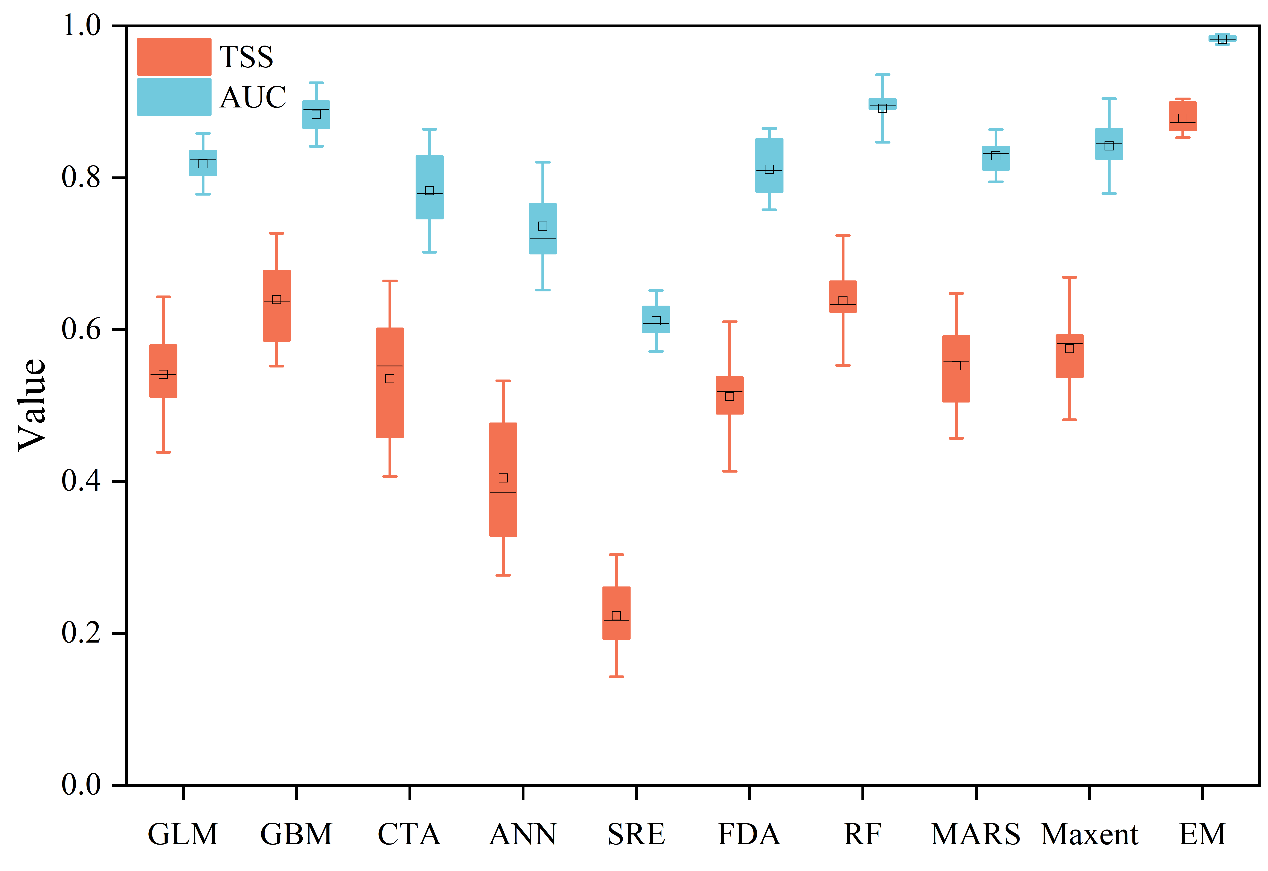
**

**Fig. S2. Accuracy assessment of the independent model of *Rhombomys opimus* potential distribution**

**
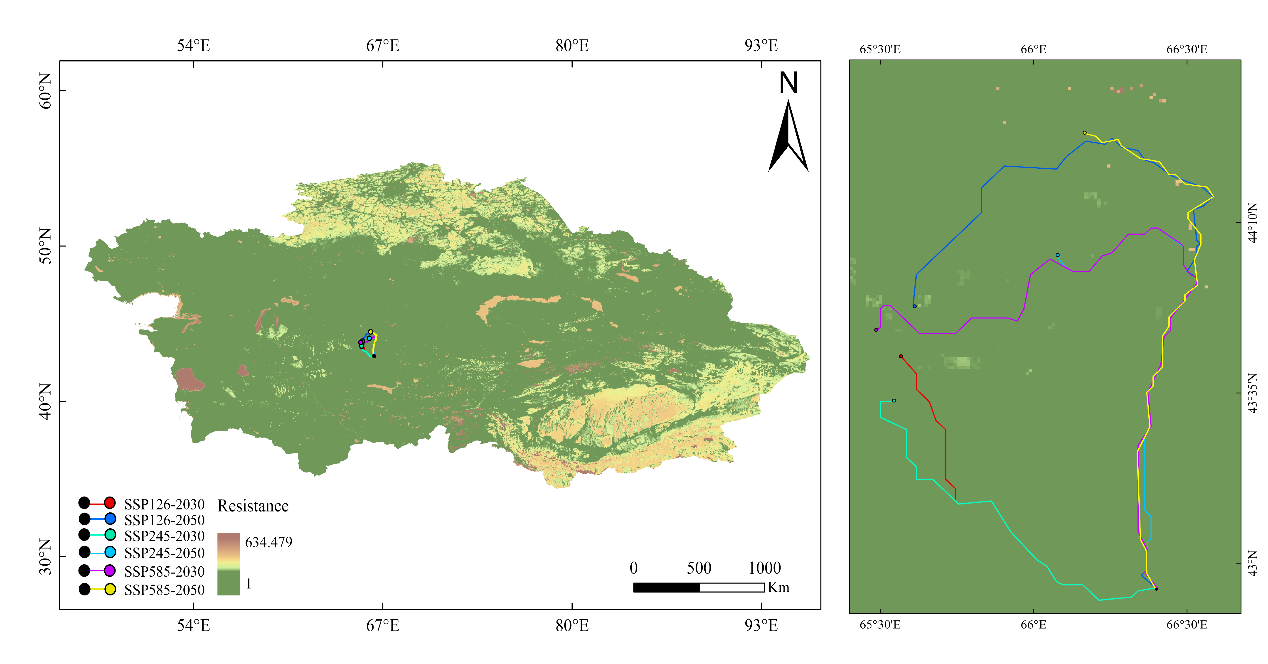
**

**Fig. S3. Potential migration pathways in suitable areas of *Rhombomys opimus* under SSP126, SSP245, and SSP585**
